# Consequences of centralized healthcare systems: changing role and autonomy of hospital managers – insights from a Hungarian case

**DOI:** 10.1108/JHOM-08-2024-0358

**Published:** 2025-02-28

**Authors:** Eva Krenyacz, Eva Erika Revesz

**Affiliations:** Corvinus University of Budapest, Budapest, Hungary

**Keywords:** Centralization, Recentralization, Hospital, Manager, Autonomy, Decision making

## Abstract

**Purpose:**

The objective of this paper is to investigate how top managers in public healthcare interpret and perceive their autonomy within a highly centralized system and how their roles and attitudes have evolved in response to centralization.

**Design/methodology/approach:**

The research examines how increased centralization and reduced organizational autonomy affect decision-making in hospitals, employing qualitative analysis through in-depth interviews with top managers. The study collected and analyzed data from 15 hospital managers in year 2015 and 2022 (eight interviews each year, one person interviewed twice), to capture changes following significant centralization efforts and the effects of the pandemic and health sector reforms.

**Findings:**

Centralization has reduced financial and operational managerial autonomy for many institutions, leading to delays in decision-making, especially in financial matters and has also brought significant administrative and reporting burdens. Despite this, hospital managers reported retaining some professional autonomy in developing and managing their service portfolios, but this autonomy is primarily operational rather than strategic and is limited by financial constraints.

**Research limitations/implications:**

This study examines the Hungarian healthcare system, influenced by unique political context, which also presents a methodological limitation concerning the transferability of findings.

**Practical implications:**

Hospital top managers’ professional autonomy is often obscured by heavy administrative and financial pressures; thus, enhancing their strategic mindset is essential.

**Social implications:**

Policymakers should adopt a comprehensive perspective in hospital maintaining, with a particular focus on balancing financial and medical perspectives.

**Originality/value:**

The paper focuses on an under-explored area: the organizational autonomy of hospital top management in the context of centralization efforts, delivering a message to both hospital managers and policymakers by emphasizing organizational aspects.

## 1. Introduction

After a long period of decentralization efforts, a potential new paradigm of (re)centralization emerged in the healthcare sector, and the role of the central government had begun to strengthen in several countries (Saltman, 2008; Terlizzi, 2019a). The recentralization of health systems that were previously decentralized has already occurred in Italy, Denmark (Terlizzi, 2019b), Brazil and Spain (Ferreira do Vale, 2022). Poland is currently considering robust plans for recentralization (Dubas-Jakóbczyk *et al*., 2023).

Until 2010, decentralized mechanisms played an important role in coordination of various sectors in Hungary, however after 2010, the centralization of the health care sector, along with other sectors, became prominent. This shift resulted in a perceived reduction in organizational autonomy, as communicated by top managers, although the literature does not uniformly categorize these changes as centralization (public hospitals no longer belong to local governments and are run by a bureau of central government (Kornai, 2015), certain periods have been characterised more by concentration of care and efficiency gains. These developments have undoubtedly impacted the autonomy of top managers, albeit in varied perceptions and experiences.

In Hungary, the concentration of healthcare services had already been present to some extent in the 1990s, with centralization intensifying a decade before the COVID-19 pandemic and further accelerating during it. This intensification included the enactment of legislation (through decree governance) that bypassed organizational managers, such as changes in physicians’ employment status, the reorganization of on-call services and the outsourcing of technical staff. As a result, Hungary did not experience the phenomenon of intergovernmental conflict (over the balance of authority between central and regional governments, as in Italy (Salvati, 2022)) and not resulted in a fragmented response to the crisis. Nevertheless, these changes had a profound impact on organizational operations, including leadership, control and strategy.

This paper explores the concept of autonomy as defined in the public management and health care context, viewed through the perspectives of top managers. We specifically analyze the Hungarian healthcare system, aiming to draw general conclusions applicable to other healthcare systems, despite Hungary’s unique political structure (Ádám and Csaba, 2022; Hajnal, 2020). The Hungarian case provides a valuable opportunity to examine how (re-)centralization efforts influence the role of hospital management and perceived organizational autonomy. The objective of this paper is to investigate how top managers in public healthcare interpret and perceive their autonomy within a highly centralized system and how their roles and attitudes have evolved in response to centralization.

## 2. Conceptual background: centralization and organizational autonomy in the public sector

Our paper analyses the changes of organizational autonomy in the context of (re-)centralization. First of all, we will define the main concepts of the paper. Centralization is considered a change that affects the decision-making, control and instruction competencies by partially or wholly transferring them to an upper level of the administrative hierarchy (Hutchcroft, 2001). Centralization can result in a more uniform and consistent approach to governance but may also lead to slower response times and reduced responsiveness to local needs.

In the healthcare sector, centralization could involve the reorganization of health services into fewer specialized units to improve quality of care and reduce costs (Vonlanthen *et al.*, 2018). Providing services in fewer units can be described by the concept of concentration. The merger of units that were previously functioning as distinct organizations does not inherently mean transferring of control and decision-making responsibilities to a higher hierarchical level. A concentration of services would result if the merged organizations operated at the same hierarchical level; conversely, centralization would ensue if some decision-making, control and instruction competencies were to be transferred to a higher level of hierarchy concurrently.

Our research also considers the notion of organizational autonomy to be significant, alongside the concepts of concentration and centralization. The term “autonomy” frequently serves as a conceptual umbrella that covers a variety of other concepts. For instance, legal, financial and managerial autonomy can be differentiated, with each term emphasizing a distinct facet of the notion.

The central concept of our research is organizational autonomy, defined as follows: “a unit’s extent of actual collective decision rights over its resource orchestration decisions and actions vis-à-vis its parent organization which has the formal power to grant this autonomy.” (Arregle *et al*., 2023, p. 98). In terms of the degree of organizational autonomy, there can be a substantial difference in the perception of the subordinate and superordinate manager, and some authors therefore propose the concept of “assumed” organizational autonomy, which captures the different perspectives and perceptions of superior and subordinate units (Cavanagh *et al.*, 2023).

Organizational autonomy is relative and limited, and this is especially true in the public sector (including the healthcare sector), where the delivery of public services and organizational operations are subject to numerous legal, financial and structural constraints. Verhoest *et al.* (2004) examined organizational autonomy in the public sector from two perspectives: firstly, as the level of decision-making competency (discretion) of an organization, secondly, as the exemption of constraints on decision-making competencies (Verhoest *et al.*, 2004). Autonomy in decision-making competencies involves both managerial and policy autonomy. Managerial autonomy refers to an agency’s discretion in managing inputs (including financial management, human resources and other production factors), while policy autonomy refers to its ability to make decisions about the content and structure of primary processes, output and outcomes (Roness *et al.*, 2008). Both managerial and policy autonomy can be understood at operational and strategic levels. The operational managerial autonomy, for instance, means that the organization may take managerial decisions concerning, for example financial transactions within strict procedures/rules set by a superior institution. This interpretation is summarized in the Table 1.

**Table 1:** Different aspects of organizational autonomy based on Verhoest *et al*. (2004)

Types of autonomy		Operational	Strategic
Managerial	The choice and use of inputs	At the level of individual transactions or employees	At the level of the general rules and criteria
Policy	Decide on certain aspects of the implementation of policies and outcomes	Prioritization of activities	The quality or quantity of outputs, outcomes

In their interpretation of organizational autonomy as the exemption of constraints on decision-making competencies, Verhoest *et al*. distinguish the following categories: structural, financial, legal and interventional autonomy (Verhoest *et al.*, 2004). Structural autonomy refers to who appoints and evaluates the head of a public organization. Low level of structural autonomy are seen when the head of the organization is designated by the central government and is directly accountable to a central bureau. A higher degree of structural autonomy is present when an elected board appoints and assesses the head of the organization.

The extent of financial autonomy depends on the composition of organization’s financial resources (whether there is an other source of funding in addition to the central government) and the scope for financial management (whether there is profit responsibility at organizational level). In the context of legal autonomy, we evaluate whether the organization possesses a distinct legal personality and, if so, whether it is subject to public or private law. Lastly, interventional autonomy pertains to the degree to which the central government regulates the organization’s operations in a detailed and strict manner, the organization’s reporting obligations and the potential consequences of operating outside the rules.

The analysis of the impact of centralization on the functioning of hospitals and the transformation of top management work will be enhanced by an understanding of these dimensions of organizational autonomy.

## 3. Historical overview and political context of Hungarian healthcare system

### 3.1 Evolution of ownership relations in the hospital sector from the soviet era

In the post-socialist countries of Central and Eastern Europe, the era of democratic transition and EU accession has seen significant decentralization initiatives across various sectors, including regional policy, education and healthcare. However, in the past decade, decentralization has diminished from the policy agenda in the post-socialist Central and Eastern European reginon, including Hungary (Loewen, 2018).

**Figure 1:**
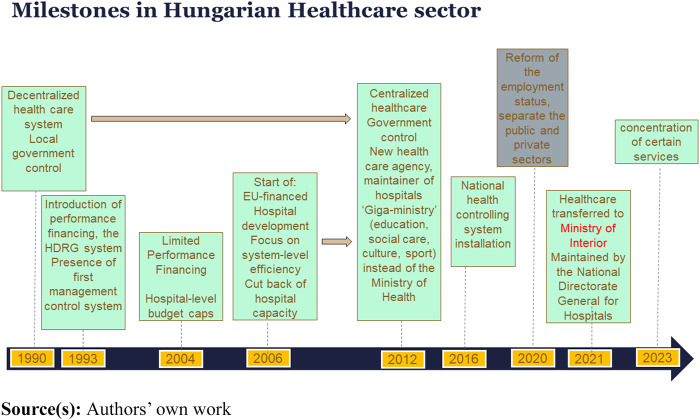
Milestones in Hungarian healthcare sector in the last four decades

As a result of Hungarian healthcare reforms in the 1990s, central government control was replaced by decentralized operation and local municipalities largely became the dominant providers of healthcare services. This arrangement persisted with minor adjustments until 2012, when formerly municipally-owned hospitals were reintegrated into state ownership, often accompanied by their outpatient facilities. These institutions were subsequently consolidated under a centralized organizational and professional management institution, named the National Institute for Quality and Organizational Development in Healthcare and Medicines (Halmosi, 2022). This institution is supplied with budgetary, regulatory, property management, centralized procurement and service management responsibilities. Later, in 2015, the institution’s successor with reduced powers and responsibilities, was established (National Health Provider Centre). In parallel, the tasks of the Ministry of Health have been integrated with the Ministry of National Resources in other sectors, such as sports, culture, social sector, public education and higher education. In this process, numerous hospital top manager positions were opened for applications nationwide. This event significantly influenced the top managers’ willingness to work in healthcare, which is a determining factor from the point of view of our research. In 2021, the healthcare subsystem was transferred again, but this time to the Ministry of the Interior (since the socialist regime changes, it has been maybe the most stable, unchangeable ministry). The central agency of health care has been renamed to National Directorate General for Hospitals, even though its functions have not changed, its responsibilities are monitoring the functioning of the healthcare system, providing the basis for strategic government decisions and contributing to the development of a new, integrated and transparent national healthcare governance system (see Figure 1).

A consequence of the structural modifications is that a number of active hospital beds nationwide has decreased, and a shift towards chronic, day-care and outpatient facilities have ensued. According to the latest data, a total of 158 healthcare institutions provided services financed by social insurance, with the distribution and size of their ownership, delineated in Table 2.

**Table 2:** Institutional data funded by national social insurance

Institutions by maintenance	2023		2014		2011		2003	
	No of instutions	No of beds	No of inst.	No of beds	No of inst.	No of beds	No of inst.	No of beds
Healthcare institutions maintained by local government	15	73	14	83	112	55,404	117	60,747
Healthcare institutions maintained by central government	83	47,905	100	58,744	15	6,492	26	9,846
University	4	10,407	4	7,366	4	7,359	4	7,446
Enterprises	25	1,567	24	1,577	12	187	1	68
Religious institutions	9	1,843	9	1,452	7	1,463	9	1,609
Other	22	636	16	401	21	592	19	526
Hungarian prison service	–	–	1	311	2	608	2	608
*Total*	*158*	*62,431*	*168*	*69,934*	*173*	*72,105*	*178*	*80,850*

**Source(s):** Authors’ own work

Changes during this era were not only reflected in organizational transformation but the healthcare structure became more concentrated. The number of hospitals maintained by both central and local governments has decreased by more than 30% in terms of both the number of institutions and bed capacity. Within this reduction, local governments have transferred most of their hospitals to the central government, leaving them with only 15 institutions, primarily focused on outpatient care. In parallel, privately-owned organizations have been granted greater operational flexibility, resulting in an increased market share.

### 3.2 The financing and operating of services provided on a social security basis

Healthcare services are financed by the National Health Insurance Fund for Hungary (hereon as NHIF) as a central purchaser of services. The financing of the out-patient care and the in-patient hospital care are based on performance funding) (Orosz and Burns, 2000), but exclusively up to a budget cap. The performance-based financing system was introduced in 1993, based on the American model, converted to Hungarian specificities. Throughout the years hospitals got to know financing extensively, and to increase their income they introduced over-coding. However, this method had to be limited (Kornai, 2009), so in 2004 a finance cap was set – the performance-volume limit (hereon as PVL). The essence of PVLs is that a limitation is put on the annual performance of hospitals, and the NHIF finances care services over the limit only partially, or not at all (Krenyácz, 2020). The PVL is a defined eligible output volume for outpatient care and active inpatient care for service per year, on a monthly basis. During the COVID-19 pandemic, social security funding was allocated based on the average funding from a preceding period rather than on actual performance metrics. At national conferences, experts highlighted that hospitals have faced significant challenges in transitioning back to a performance-based funding model. Despite these challenges, the government has reintroduced performance-based funding without adjusting for inflation, VAT, overhead costs or wage increases, opting instead to provide these adjustments as supplementary funding. This approach suggests that the government may not fully endorse performance-based funding, although the underlying reasons for this remain unclear—whether they are technical, professional or political.

The outsourcing of hospital support services, specifically the centralization of certain activities, has been an ongoing process. Last year, the technical services were consolidated under a single institution, from which hospitals now procure services such as heating, plumbing and maintenance. The government has repeatedly proposed the centralization of economic activities, such as accounting and payroll, although these plans have not yet been implemented. For decades, the procurement of materials, chemicals and pharmaceuticals has been conducted through centralized public procurement processes.

In terms of professional activities, smaller institutions are increasingly being integrated as satellite sites of larger institutions, as demonstrated in Table 1. Although it remains uncertain how these “simplified” hospitals will achieve the proposed HR cost reduction.

## 4. Methodology

The primary objective of our research is to understand how the extension of centralization and the reduction of autonomy influence organizational decision-making and the roles of managers in hospitals. We used qualitative analysis based on in-depth personal interviews to address the question. The data were collected over two-time periods, enabling us to gain insight to the movements and changes of the past decade. The two rounds of interviews occurred in 2015, immediately following the first powerful centralization, and in 2022, after the pandemic crisis and healthcare employee reform. In the first round, eight interviews were conducted with top managers of hospitals and with one consultant, who has access to hospital accounting and controlling data. During the second data collection, eight additional interviews were done, including one participant from the first data collection. All the interviewees (refer to Table 3 below.) have more decades of work experience in the health sector. The interviews were conducted in a semi-structured format. We began with open-ended questions, focusing not on the system or centralization, but rather on the role of management and leadership. We also explored the expectations coming from external sources and the scope of their managerial responsibilities. The questions relevant to this study were outlined in the interview guide:

What governance structures and management approaches characterize the hospital's administration? For example team-based management, executive leadership, external support mechanisms, etc.

How do hospital managers employ control mechanisms and management information systems? (What is the impact of these practices on organizational operations and decision-making processes?)

What decision-support tools and methodologies are implemented in the hospital? (To what extent do these tools enable flexibility within the constraints faced by public hospitals?)

How are financial and economic considerations incorporated into clinical decision-making processes?

What expectations does the leader experience in their managerial role, and from whom or where do these expectations originate? (How can these expectations be characterized? How does the leader perceive and navigate the entirety of these demands?)

In which areas of their managerial work does the leader feel tension most acutely, and under what circumstances? (Do conflicting expectations pose challenges, and if so, how are these addressed?)

**Table 3:** Characteristics of the interviewees with the year of data collection

Interviewee code	Position/role	Type of institute	Year of data collection
A (1,2)	Medical director	Central hospital	2015, 2022
B	Director general	Urban hospital with national competence	2015
C	Director general	Central hospital	2015
D	Chief financial officer	Urban hospital	2015
E	Expert (consultant) of health management		2015
F	Director general	Hospital specializing in rheumatological and psoriasis treatment	2015
G	Chief financial officer	University hospital	2015
H	Expert (consultant) of health management		2015
I	Director of nursing	Hospital specializing in pulmonary medicine	2022
J	Director general	Managed hospital by central hospital	2022
K	Deputy director-general for strategy	Central hospital	2022
L	Director general	Urban hospital	2022
M	Director general	Urban hospital	2022
N	Director general	Hospital specializing in pulmonary medicine	2022
O	Director general	Urban hospital	2022

**Source(s):** Authors’ own work

The interviewers are independent academic researchers and educators with experience in health administration, management and non-profit organizations. They have formerly served as experts but are neither hospital employees, nor affiliated with the surveyed institutions. The interviews were conducted either in person or via the Zoom platform, depending on the interviewees’ preferences. All conversations were audio-recorded (with the participants’ consent) and transcribed using Alrite software, which is optimized for the Hungarian language but requires follow-up work.

The interviews were analyzed using thematic analysis (Strauss and Corbin, 1998), we began the multi-level coding process with open coding, attempting to identify as many themes as possible, then we looked for relationships between the codes, grouping and categorizing them. The final code structure is the following: system (financing, limitation, professional element, as protocol or waiting list, co-payment), decision-making (resources, data, competencies, processes, output and portfolio, limits), human resource (manager, staff, interest, attitudes, perceptions), strategy and long-term perspectives. When coding the interviews, we also treated the years separately to be able to draw conclusions about the changes between the two periods, and to examine the effects of activities, feelings and attitudes.

## 5. Findings

When presenting the research findings, a detailed discussion is provided on the common categories that emerged during the coding process described in the methodology. Specifically, the analysis focuses on how the interviewed hospital managers interpret and perceive autonomy, how they discuss centralization and decentralization, how they link changes in their roles to changes in the system and finally, what emotions, feelings and attitudes were expressed during the interviews.

### 5.1 Interpretation of “autonomy” in hospitals

Assumed organizational autonomy is determined by what hospital managers can decide, where and how decisions are made. Most respondents mentioned a consistent decline in financial autonomy and highlighted the presence of rigorous financial supervision. The central authority expects that hospitals would maintain optimal financial balance and abstain from creating additional debt. One consequence of performance-based funding is that managers frequently conduct weekly or even daily reviews of patient turnover and revenue data. In this regard, top managers place significant importance on the utilization of this information when making daily decisions.

Every morning, the day begins with patient turnover on the managers’ desks. Each day. We don’t look at it once a month, but rather every day. I notice the empty beds, the various departments, and how they operate. And I can see how much funding is predicted to be available each month. And where are we on the monthly budget today? (interview N)

Concerning expenditures, managers must adhere to a variety of rules, including those governing the compensation of physicians and nurses as well as public procurement regulations for purchases. These characteristics have not been substantially altered by centralization. As a consequence of mergers, certain hospitals have lost their economic autonomy. An interviewee elaborated on how a hospital merger had altered the economic maneuvering space of the organization.

We’ve been a partner hospital since 2021. Despite our professional independence, we are economically dependent. Our economic and technical autonomy has been weakened in this regard, and every minute I must ask for my own money and whether I can send up the authorization to use it. This represents a major operating delay in the system. Sometimes it takes two months for the approval to be returned … (interview N)

Beyond financial resources, the most important resource in healthcare is highly qualified people. Managers can hire physicians, nurses and support staff for hospitals, but salaries for physicians and nurses are fixed by law, and support staff are typically paid significantly lower wages than comparable positions in the business sector. The majority of interviewees stated that it is difficult to find staff in the administrative and nursing field and the turnover is high. These characteristics lead to a reduction of managerial autonomy at the operational level.

Many people have left the healthcare system and are unlikely to return due to new employment regulation. So, we lost thirteen nurses back then, and we still can’t replace them to this day. (interview N)

In addition to managerial and financial autonomy, several interviewees also referred to policy autonomy. Respondents talked about the flexibility that managers have in developing the portfolio of professional services provided by hospitals, a decision that the central authority has limited influence over. Nevertheless, a key barrier to professional development of hospitals is the insufficient availability of financial resources.

No one interferes with the profession, and they should not …. In Hungary, there is freedom to practise medicine. Of course, the funding is a framework for this (interview A2)

Overall, according to the interviews, hospital managers reported a stricter control on the use of resources in recent years, in relation to the shift towards centralization, and a corresponding increase in reporting responsibilities. The implementation of professional activities and the prioritizing of services did not show any notable alterations in operational policy autonomy.

### 5.2 Systemic elements in managerial thinking: centralization, concentration and de- or recentralization

Exploring the organizational autonomy in hospitals, centralization and concentration of healthcare from a managerial perspective provides valuable insights. Managers interpret the health system by considering (de)centralization, financing and protocols while dealing with the challenge of maintaining an economic viable institution in a dynamic environment. The changing environment is generated by not just unpredictable external environment (inflation, pandemic) but by the owner of the hospitals themselves, who ask meaningless and immediate data requests. An interviewee mentions that “we are not always able to affect our surroundings, leading to uncertainty about what is happening and why” (interview K). One explanation for it is that the hospital cannot be operated economically well, necessitating the fulfilment of financial, professional and protocol requirements, everything’ (interview L). Consequently, there is “a desperate shortage in the sector” (interview I, H), “an escalating social security fund over a 5–8-year period” (interview H) and the government sees the solution in keeping everything under control financially. Overall, these are “very depressing for a director” (interview C).

#### 5.2.1 Decentralization: age of local government

During the retrospective analysis of **decentralization** (pertaining to the era of local governance), hospital managers form a positive image, primarily emphasizing the organization (“we truly operated like a big family, always finding solutions to problems” – interview L) and communication of managerial tasks, as well as existence of reward systems.

We operated with a sense of freedom. I get the direction, have followed it, but I had several chances to go further and implement my ideas. (interview L)

Despite being aware that the decentralized era was part of a political power play, managers still perceive it as “peacetime” (interview E), primarily because expectations were clear, responsibility was closer and consequences were more evident.

We really operated like one big family, always finding a way to solve the problem. (interview L)

As the director of the hospital under municipal management, I was fully aware that I could end up in debt or face ruin … If I will, services will stop; if services stop, there will be a scandal; if there’s a scandal, I'll be a front-page story; if I'm a front-page story, I'll be removed from my position, and so on, you know … (interview H)

#### 5.2.2 Centralization and concentration of care: age of state government ownership

Centralization (in Hungarian healthcare system) aims for the concentration of resources and the integration of care services; however, according to the interpretations of the interviewees, typically centralization restricts economic and financial opportunities while not impeding formulating professional portfolios and expertise. Based on these statements regarding (re-)centralization, four key categories emerge in characterizing their managerial work: a “fire-fighting” approach to management, a financial focus with less professional emphasis at the top management level, state control and a medical professional openness at lower levels.

##### 5.2.2.1 Managing in a “fire-fighting nature” – as reason of frustration

The managers feel that they need to act immediately to the problems that emerge, and they have ad-hoc reporting obligations to fulfill every request from the owner.

Your room for maneuver has increasingly diminished; you always had to solve problems—what isn’t there, what’s missing, what should be done, what should be obtained. It was always about troubleshooting, but logically you couldn't resolve it. (interview L)

Since I’ve been a hospital director, I’ve been putting out fires; it’s almost impossible to build. Something is always coming up. In an ideal society, health care is independent of politics, and everyone supports it. (interview A2)

##### 5.2.2.2 System requires financial-focused management work

Some hospital managers emphasize the professional aspects, but typically, their time and thoughts are predominantly occupied by financially focused work. They do not interfere in the professional aspects, yet they also do not have much energy left for them. “There were strict rules that had to be followed. Clearly, there was still some flexibility within the profession. Economically, not so much, because everything was subject to public procurement—how to acquire, where it could come from, where it could go. I mean, in terms of finances, you didn’t have much influence. However, you still retained control over the medical profession. There, you could still bring into the hospital these little provisions, organize training sessions, submit applications in the field, set up the health development office. Perhaps this palette could be expanded. It was the economic aspect that became restrictive. (The interviewee H spoke in the past tense because they had resigned and returned to working in their medical profession.)”

Hospitals plan, decide and operate based on revenue maximization, but their managers mentioned that

Monitoring is ongoing on the revenue side (interview A1)

With ‘normal operation’, the loss is 10–15%, but of this loss, 5–8% could be solved. However, it entails significant struggle, pain, and sacrifice. Should I, as a hospital director, then undertake this? (interview E)

The financial focus entails regulation, administration, supplemented by continuous monitoring of financial revenues and managing debts. Moreover, when nationalization and centralization occurred, the maintaining institution was established, then every consolidation began with its first sentence: “Does the hospital have any outstanding debts over 60 days? If not, the maintaining institution does not deal with them.” (interview E) The maintaining institution prioritizes debt and subordinates everything else to it. The managers are critical of the maintaining entity and view centralized operations as very strict, regulated and accountability-focused system, however, they do not perceive its supportive function, which would make healthcare delivery more flexible and patient-centered.

On the other side of the table, there isn’t really anyone sitting who could analyze or intervene, not to mention the implementation of financial management measures. (interview H)

##### 5.2.2.3 State oversight and control at all levels

In terms of central hospital management, there is a strong emphasis of the State on regulating all processes through information systems. This approach includes the implementation of a uniform chart of accounts and controlling regulations. The general sentiment towards these accounting regulations is cautiously optimistic, “it could be good: that if there is a single owner overseeing multiple institutions, such standardization could be beneficial, as it allows for easier comparison between different entities” (interview A1).

The literature also highlights that centralization can lead to slower decisions, and this is reflected in the interviews. A recurring complaint is the high demand for data to be provided to the central office, usually on short notice “awful data requests from central office, awful administrative burden” (interview C).Nevertheless, the reporting process is intensive, with institutions required to provide constant updates on their performance and status. However, this reporting seems to function in one direction only, “we constantly had to report on how we were doing, where we stood, so we were continuously monitored” without any feedback’ (interview L), as there is a notable absence of feedback, leaving institutions feeling continuously monitored without any corresponding support or guidance.

Concerning the payment of employees, every hospital staff payment must be accepted by the state.

Even though we are professionally independent, it is a bit of a dependency … we have the economic dependency. Our economic and technical autonomy has been taken away in such a way that I must ask every minute for my own money to spend it. So, this is a significant operational slowdown in the system. Sometimes it’s two months before that sighting comes back and you can spend your own money to order that order. (interview N)

The managers are not only tied in the financial area, but also have difficulties in professional human resource planning, although these will be a problem in very specific cases for example “The employment relationship of doctors with the operator allows the right to reassign employees to another institution, excluding the top management” (interview I) or the reorganization of the central on-call service. There is resistance to the system, but reasonable professional decisions are appreciated, although it would be better to make them from the bottom up.

Finally, we mention the most serious influencing factor for hospital managers (and for the system) that “every hospital manager” employment contract allows for termination without cause at any time’ (interview J). This is perhaps the greatest source of uncertainty in the lives of managers and should be borne in mind when interpreting chapter 5.3.

##### 5.2.2.4 Medical professional openness

Managers perceive autonomy across the entire professional spectrum, as illustrated by the quotes below. However, they do not interpret these perceptions as indicative of autonomy; that is, these elements did not surface in their definitions of personal autonomy.

They supported me professionally, so if I wanted to introduce anything new, they never vetoed it. (interview L)

I still retained control over the profession. There, you could still bring into the hospital these little provisions, organize training sessions, submit applications in the field, set up the health development office. (interview L)

However, this professional autonomy is a privilege of doctors, and it can be achieved by collaborating with them and, most importantly, motivating them. Currently, the physicians cannot be financially motivated because of the lack of financial autonomy and the financial constraints.

This is further complemented by the interviewee’s claim, who observed that performance management systems were not implemented effectively even in the past and it was true not only on organizational but on system level. For example, “we were able to give bonuses to everyone, not based on performance or incentives, but everyone received a large amount” (interview L). This applies to the operation of the system as well; even with debt consolidation, they don’t reward those who perform well. “If you don’t have debt, then be quiet, you won’t get anything.” (interview E).

### 5.3 Leadership role transformation in systemic change: interactions and connections, feelings and attitudes

In general, the study found that respondents see a decrease in organizational autonomy as compared to the decentralized system. The level of managerial operational autonomy has been lowered, there is strict financial supervision over hospital management on both the revenue and expenditure sides, and the human resource management toolbox has been limited. The use of individual performance evaluation and incentive tools is almost non-existent in the centralized system. Although doctors’ salaries have increased significantly in recent years, those of nurses and support staff have increased much less.

Regarding policy autonomy, which refers to the ability of hospital managers to determine the professional processes and service portfolio, the interviewees believe that they have considerable room for maneuver. This autonomy is functional in nature rather than strategic. And the decision-making authority is rarely utilized by them, primarily for financial reasons. Additionally, several interviewees highlighted the fact that a considerable percentage of health administrators lack managerial competencies and, as a result, are incapable of implementing policy autonomy.

70% of managers cannot lead–this is my belief–not because they are not good people, but because they have no idea what is expected of them, they do not know basic (management-organizational) techniques, methods and tools (interview F)

There are few qualified, agile nursing directors with an overview of the hospital. (interview A2)

Your room for maneuver has increasingly diminished; you always had to solve problems—what isn’t there, what's missing, what should be done, what should be obtained. It was always about troubleshooting, but logically you couldn’t resolve it. (interview B)

The manager’s task is building the right team, and in this regard the manager’s expectations have not changed between the two periods, but rather the awareness has increased that a good team is necessary for quality patient care. Building a strong team is a leadership challenge, and interviewees highlighted the difficulty of finding the right members for both the management team and the teams involved in professional operations.

It is hard to build a good management team. (interview M)

… and I was able to put together a very good team of doctors, which made a big difference. (interview L)

Unfortunately, managers are finding it increasingly difficult and challenging to fulfil the requirements, not only because of central regulations but also because of changes in the workforce.

There is a shortage of nursing staff: Professionals are leaving, but it’s because of wages’ (interview K) it is difficult to find workers … a department is short a team of people, about sixteen of them (interview I)

a lot of people have dropped out of the health system, who are not coming back into the health system, because of the change in legislation, and we still can’t get them back in. (interview N)

Beyond considerations of funding and external environmental factors, interviewees were more inclined to discuss their professional endeavors and long-term strategic thinking. They recognize the significance of adopting a strategic perspective and appreciate its importance. However, they also express concerns about the lack of sufficient time and guidance from the governing bodies or ownership, which they believe hinders the effective implementation of these strategies.

We have a professional development concept, so we also look to the future, but in proportion to the present, and for decisions in the present we usually use the figures of the recent past. (interview K)

We do not take instructions from above on where to go and what to do. We have our own professional development plan, our own concept, and we obviously update it and try to present it to decision-makers. (interview C)

New care has been brought into the system. We restructured, rationalized the nursing activities a little bit, rationalized the departments, so we did some good things professionally that moved the institution forward, so our reputation grew, our image got a little bit better. (interview L)

Overall, managers are finding it particularly difficult and frustrating to keep up with the regulatory environment, to comply with the huge amount of data (“should have been submitted yesterday”) and to cope with rapid and increasingly frequent changes.

## 6. Discussion

The primary objective of the Hungarian government in centralizing or concentrating health care is clear: to enhance the economic efficiency of health care and to effectively distribute the required resources to the various services. Comprehensive consensus exists that the system is financially unsustainable due to insufficient amount and allocation of resources by the state. The central government is trying to decrease the size of the health system by means of centralization, concentration, expansion of public procurement and outsourcing/coordination of similar operations. This is done to maximize economies of scale and enhance resource utilization efficiency. However, health care is a highly complex area of power, and the interests and counter-interests fail to align for the system to function ideally. Consequently, all of this led to an overly regulated and very rigid system.

In the process of centralization, top managers encounter challenges in adapting to the continuous and unpredictable changes in external circumstances and the necessity of quick responses. While the actual value of hospital funding has declined over the past 5–8 years, hospitals are now required to adhere to a variety of frequently changing requirements, including minimum standards, financial requirements, public procurement rules and professional protocols. Consequently, it has become increasingly challenging to operate hospitals economically.

The centralization efforts have resulted in a reduction of financial and operational managerial autonomy for numerous institutions. This has culminated in delays in operational decision-making, especially in financial affairs, as approvals from central authorities are now necessary. In our case, centralization is accompanied by a substantial administrative and reporting burden for hospitals. A certain amount of this is attributable to the fulfillment of obligations associated with stricter regulation, while another part is due to the enhanced information needs of centralized operations.

The transformation of leadership roles was also reported by interviewees. It was confirmed that “governments” attempts at more managerial approaches to public service provision in reality add new ex post controls without reducing the old ones’ (Öberg and Wockelberg, 2021). Centralization has resulted in a mainly top-down way of communication in Hungary, and hospital managers feel that they are not involved in decisions. This shifts the focus of top managers’ work from thinking about strategy and concept formulation, unified organizational functioning to fragmented, reactive, task-focused thinking.

Despite the centralization, hospital managers reported retaining some degree of professional (policy) autonomy, particularly in developing and managing the hospital’s service portfolio. However, this autonomy is largely operational rather than strategic, severely limited by financial constraints. Furthermore, they feel that they do not receive support from the superior authority to implement their professional visions.

Many respondents contrast the current centralized system with the earlier decentralized system, where hospitals operated under local government control. Some managers nostalgically recalled this era as one of greater autonomy, clearer expectations, despite the greater financial responsibilities. There was a significant increase in frustration and stress levels among respondents. Some top managers have left or are thinking about leaving their careers or are trying to exit state-run hospitals.

The centralization efforts observed in the health sector do not inherently lead to the over-regulated and inflexible system that has emerged in Hungary. Terlizzi argued in her book that “the distinction between decentralized and centralized health systems is a matter of degree rather than a fully-fledged dichotomy” (Terlizzi, 2019b, p. 170). There are many ways of centralization, including the reallocation of responsibilities across different layers of government and reorganization of governance, control and reporting processes. This is highly dependent on the institutional set-up and political context of the country. Centralization is diminishing perceived organizational autonomy, significantly changing the role of and expectations towards hospital managers; therefore, it is crucial to monitor and assess the implications of centralization on hospital operations.

## 7. Limitations and future research

The process and findings of our research should be considered with the usual limitations of interview-based studies (Kvale, 2007). The reliability, validity and transferability of results are essential in understanding and evaluating the limits of qualitative research. Reliability refers to the consistency and trustworthiness of research findings. This topic relates to the transcribing and analysis of interviews, specifically on the consistency of results produced by different transcribers and analysts. All interviews were audio-recorded with the consent of the interviewees, and the transcript generated using Alrite was compared to the original audio recording. Members of the research team participated in the analysis, and findings were checked continuously.

Validity means whether an interview-based study investigates the intended research objectives. To ensure the research’s validity, the interviewer and interpretation bias were mitigated by include two members of the research team in each interview, and the interpretation process was executed in multiple rounds with peer validation. The sample size consisted of 15 hospital managers, and in an interview-based research it is indeed difficult to determine the appropriate number of interviews. In the next research phase, we plan to continue the data collection and include more interviewees. Expanding the sample to include participants beyond state-owned hospitals may represent a significant direction for future research.

Interview-based research is context-specific and has limited applicability in other contexts. This study aims to offer a rich contextual description to facilitate the identification of additional contexts where the research findings might have relevance.

## 8. Conclusion

This paper investigates how top managers in public healthcare interpret and perceive their autonomy within a highly centralized system and how their roles and attitudes have evolved in response to centralization. For this purpose, we have briefly reviewed the literature on organizational autonomy, which is not essentially drawn from healthcare literature, rather from public management. Then, with qualitative analysis through in-depth interviews we collected and analyzed data from the years 2015 and 2022.

The centralization of Hungary’s healthcare system has a significant impact on hospital management, particularly in terms of organizational autonomy. While centralization has brought about greater uniformity and control, it has also led to a reduction in the financial and managerial autonomy of hospital top managers. This has resulted in a more reactive management role, characterized by constant crisis management.

It is essential that central authorities realize the importance of maintaining a balance between control and autonomy to provide hospital top managers with the adequate support to effectively lead their institutions. This includes providing clearer guidance (instead of detailed rules), reducing administrative burdens and fostering a more collaborative relationship between central authority and hospital management.
